# Conspiracy narratives and vaccine hesitancy: a scoping review of prevalence, impact, and interventions

**DOI:** 10.1186/s12889-024-20797-y

**Published:** 2024-11-29

**Authors:** Frederike Taubert, Georg Meyer-Hoeven, Philipp Schmid, Pia Gerdes, Cornelia Betsch

**Affiliations:** 1 Institute for Planetary Health Behavior, Health Communication, University of Erfurt, Erfurt, Germany; 2 Health Communication Working Group, Implementation Research, Bernhard Nocht Institute for Tropical Medicine, Hamburg, Germany; 3 Centre for Language Studies, Radboud University Nijmegen, Nijmegen, The Netherlands

**Keywords:** Vaccination, Immunisation, Conspiracy theory, Conspiracy mentality, COVID-19, MMR, HPV, Review, Interventions

## Abstract

Believing conspiracy narratives is frequently assumed to be a major cause of vaccine hesitancy, i.e., the tendency to forgo vaccination despite its availability. In this scoping review, we synthesise and critically evaluate studies that assess i) the occurrence of vaccine-related conspiracy narratives on the internet, ii) the prevalence of belief in vaccine-related conspiracy narratives, iii) the relationship between belief in conspiracy narratives and vaccination intention or vaccination uptake, and iv) interventions that reduce the impact of conspiracy narratives on vaccination intention.

In July 2022, we conducted a literature search using three databases: PubMed, PsychInfo, and Web of Science. Following the PRISMA approach, of the 500 initially identified articles, 205 were eligible and analysed.

The majority of identified studies were conducted in Europe and North America, were published in 2021 and 2022, and investigated conspiracy narratives around the COVID-19 vaccination. The prevalence of belief in various vaccine-related conspiracy narratives varied greatly across studies, from 2 to 77%. We identified seven experimental studies investigating the effect of exposure to conspiracy narratives on vaccination intentions, of which six indicated a small negative effect. These findings are complemented by the evidence from over 100 correlative studies showing a significant negative relationship between conspiracy beliefs and vaccination intention or uptake. Additionally, the review identified interventions (e.g., social norm feedback, fact-checking labels, or prebunking) that decreased beliefs in vaccine-related conspiracy narratives and, in some cases, also increased vaccination intentions. Yet, these interventions had only small effects.

In summary, the review revealed that vaccine-related conspiracy narratives have spread to varying degrees and can influence vaccination decisions. Causal relationships between conspiracy beliefs and vaccination intentions remain underexplored. Further, the review identified a need for more research on interventions that can reduce the impact of conspiracy narratives.

**Supplementary Information:**

The online version contains supplementary material available at 10.1186/s12889-024-20797-y.

## Introduction

Vaccines are cost-effective and successful health tools that prevent vaccinated individuals from developing potentially serious illnesses and help protect entire communities by reducing the spread of pathogens [1]. In 2019, the World Health Organisation (WHO) identified vaccine hesitancy—the reluctance or refusal to vaccinate despite the availability of safe and effective vaccines [2]—as one of the top ten threats to global health [3]. On the one hand, growing vaccine hesitancy threatens to reverse progress made in tackling vaccine-preventable diseases. In fact, countries that were close to eliminating diseases like measles show a resurgence despite the availability of safe and effective vaccines [3]. On the other hand, vaccine hesitancy can hinder the containment of new pathogens like the coronavirus when people refuse vaccination [4, 5]. Thus, it is important to investigate the drivers of vaccine hesitancy to reduce their impact.

One identified driver of vaccine hesitancy is conspiracy belief [6–8]. Conspiracy beliefs are commonly defined as beliefs that multiple actors meet in secret agreement in order to achieve a hidden goal [9]. More specific, conspiracy narratives – also known as conspiracy ‘theories’ – are characterised as oppositional, describe malevolent or forbidden acts and ascribe agency to a small group of conspirators [10]. These beliefs have long been part of human interaction and occur often during societal crisis situations like pandemics [11]. During the past 20 years, interest in research on conspiracy beliefs has increased [10].

One of the most common ways to get in touch with conspiracy narratives is through social networks [12]. Since social media has become an important source for health information [13], it also plays a crucial role in the spread of anti-vaccination conspiracy narratives [14]. On Web 2.0, users are enabled to quickly develop and share any content. Thus, content from users on platforms like Facebook, Twitter, and YouTube can include false information and conspiracy narratives that can influence readers’ attitudes, e.g., their vaccine intention [15]. Moreover, a systematic review of anti-vaccination content showed that anti-vaccination posts are frequently more popular than pro-vaccination posts and that anti-vaccination accounts create their posts in a way that effectively grabs attention [14]. The state of research indicates that anti-vaccination content on Web 2.0 has the potential to increase vaccine hesitancy through the often uncontrolled spread of conspiracy narratives.

Research reveals that conspiracy beliefs can influence health behaviours and thus have serious consequences for individuals and societies [10, 16]. For example, people may refuse vaccines because they believe anti-vaccine conspiracy narratives like “vaccines are harmful, and this fact is covered up” [6]. The refusal threatens not only the individual’s health but also that of society, as it jeopardises the desired herd immunity [17].

Recently, during the COVID-19 pandemic, the relationship between conspiracy beliefs and health-related behaviours such as following safety guidelines and getting vaccinated became apparent [18]. A systematic review of literature investigating the association of conspiracy belief and vaccine willingness during the COVID-19 pandemic provides a picture consistently showing a negative relationship between the two concepts [19].

As interest in the impact of conspiracy narratives has increased in recent years, there have already been efforts to reduce their significance. But as conspiracy narratives are unfalsifiable [9], a simple counterargument against a narrative might not be enough. Refuting a conspiracy narrative could be perceived as evidence for the cover-up, and the sender of a counterargument could be perceived as an accomplice of the conspiracy [20]. Indeed, a recently published systematic review on the efficacy of interventions in reducing conspiracy beliefs indicates that only a few of the evaluated interventions had medium or strong effects [21].

### Present review

The past decade’s research has frequently addressed vaccine-related conspiracy narratives. Indeed, there are already some reviews summarising literature in one specific context, e.g., within the COVID-19 pandemic [19]. However, to the best of our knowledge, no review has summarised the literature addressing the impact of vaccine-related conspiracy narratives more broadly and across an unrestricted period. The aim of the present review was therefore to provide a better understanding of the role of conspiracy narratives with regard to individual vaccination decisions by addressing three main research questions.

First, to understand the significance of these conspiracy narratives, we need to know how widespread they are. Individuals come across conspiracy narratives on the internet, e.g., while reading posts on social media [12]. Thus, considerable research has investigated the occurrence of conspiracy narratives in various internet sources (e.g., social media). Yet, this literature provides an inconsistent picture, with articles identifying either a very low (e.g., [22]) and a very high (e.g., [23]) proportion of conspiracy narratives within the analysed content. Thus, it is worthwhile to systematically analyse this literature.

Additionally, the impact of conspiracy narratives depends on the number of people who believe them. Even if people are constantly exposed to a conspiracy narrative, it likely has no effect if nobody believes it. Here, the current state of research provides an inconsistent picture. While some articles have presented fairly low agreement ratings (e.g., [24]), others have identified higher agreement ratings on vaccine-related conspiracy narratives (e.g., [25]). In sum, to investigate the spread of vaccine-related conspiracy narratives, we focussed on literature based on two research fields. On the one hand, we aimed to summarise literature about the occurrence of vaccine-related conspiracy narratives on the internet, i.e., how present they are on the internet. Here, we mainly focussed on articles with content analyses. On the other hand, we wanted to analyse literature about the prevalence of belief in vaccine-related conspiracy narratives, i.e., how many people believe them. Most of these articles were based on correlative data.

In addition to the spread of vaccine-related conspiracy narratives, it is important to understand whether belief in these narratives actually has a causal impact on behaviour or intention. Again, the current state of research provides no consistent picture. Some experiments have indicated that exposure to conspiracy narratives influences vaccination intention (e.g., [6]), while others have not shown significant effects [7]. Moreover, a systematic review of randomised controlled trials concluded that not all investigated health misinformation had an impact on behaviours and their psychological antecedents [26]. Thus, we wanted to summarise and systematically analyse this literature and narrow the focus to the topic of vaccination. Nevertheless, because more correlative studies than experiments are available, we also considered the literature about the association between conspiracy belief and vaccination intention. Thus, we included articles that directly examined the behaviour (vaccine uptake) as well as articles that indirectly explored the behaviour through the assessment of individuals’ readiness to get vaccinated, also known as vaccination intention [27].

Lastly, the potential harm of vaccine-related conspiracy narratives could be weakened by effective interventions. Thus, we were interested in studies that have empirically tested how to minimise beliefs in conspiracy narratives and their impact on vaccination intentions. We therefore included studies that measured the impact of an intervention on conspiracy beliefs or on vaccination intention.

## Method

### Open science statement

All relevant data and materials are available online on OSF (https://osf.io/2z4et/). The materials include a dataset with all identified studies (“Review Data”), documentation about the review process (“Review Process”), and a supplemental file (“Supplement”).

### Search and selection procedure

For the literature search, we used PubMed, PsychInfo, and Web of Science, covering research from medicine, psychology, sociology, and public health.

To identify articles related to conspiracy narratives, we used the keyword conspir*. Based on a systematic review by Schmid et al. [28], we added a combination of terms to cover various aspects of vaccination. Consequently, the complete search string for all databases was “conspir* AND (vaccin* OR immuniz* OR immunis* OR inoculat* OR prevention and control)”.

**Fig. 1:**
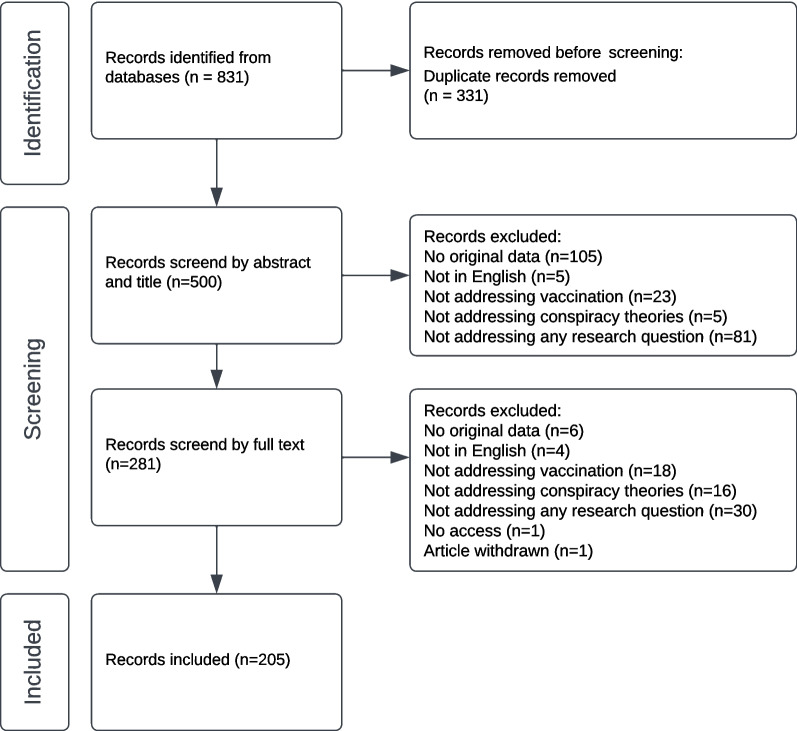
Flow Chart of selection process

The initial search was conducted from 1 – 11 July 2022. The selection procedure followed the PRISMA approach summarised in Fig. 1. First, duplicates were removed. The remaining articles were scanned by title and abstract. Afterwards, a full-text analysis was performed on the remaining articles. Here, articles were excluded according to the following a priori exclusion criteria: not addressing (human) vaccination, not addressing conspiracy narratives, not a peer-reviewed journal article, not reporting primary data (e.g., meta-analyses, reviews, comments), modelling studies, third-party observations (participants not expressing their own perspective), not published in English or German, full text not available. When the databases provided filters for type of literature (peer-reviewed) or language (English and German), these filters were used during the initial search. There was no restriction regarding publication year.

The selection was carried out by two raters (GMH and FT) who scanned the title and abstract of the first 50 articles (10% of the sample) independently. Then, their inclusion vs. exclusion ratings were compared. Since the first comparison showed a disagreement rate of 16% and a good Cohen’s kappa (k = 0.66) [29], each rater coded half of the remaining articles (for details, see “Review Process” in OSF, https://osf.io/2z4et/). For the full-text analyses, both raters scanned the first 30 articles (10% of the sample) independently to again compare and discuss their ratings. This comparison showed a disagreement rate of 30% and a moderate Cohen’s kappa (k = 0.40) (for details, see “Review Process” in OSF, https://osf.io/2z4et/). Both raters independently scanned all remaining articles in order to improve the consistency of their subsequent ratings. Finally, the complete datasets were compared. Differing ratings were first discussed among the raters, and in cases of disagreement, a third reviewer (PS) was consulted.

After the selection procedure, information from the remaining final articles was extracted and coded by two coders (FT and PG). An overview of the categories used for the coding of the extracted information is provided on OSF (https://osf.io/2z4et/).

During the categorising process, we noticed that not all narratives labelled ‘conspiracy narrative’ in the studies fulfilled all criteria of the common definition [9]. Some narratives lacked the characteristic of a malevolent intent behind the conspiracy, or, more specifically, did not explicitly refer to it. For example, in the narrative “COVID-19 vaccines will lead to infertility” [30], it can be assumed that people believe that this infertility is a consequence of a conspiracy. However, infertility could also be the result of errors without malevolent intent. Nevertheless, we decided to include all studies that claimed they had assessed conspiracy narratives.

## Results

### Identified literature

The described search strategy resulted in 831 records (Fig. 1). After removing duplicates, 500 articles underwent title and abstract screening. During the screening, 219 articles were removed after the title/abstract analysis, and 76 articles were removed based on the full-text analysis according to the exclusion criteria. Two articles could not be found. Thus, authors were contacted and asked for access, which was successful in one case. The screening procedure led to a final dataset of 205 articles.

### Descriptive analysis of articles

The identified research was conducted in different WHO regions. Most of the articles were based in Europe (88/205) and the Americas (57/205). Fewer articles were from the Western Pacific (21/234), the Eastern Mediterranean (16/205), Africa (10/205), and South-Eastern Asia (4/205). Some (8/205) included a sample with participants from more than one WHO region.

**Fig. 2:**
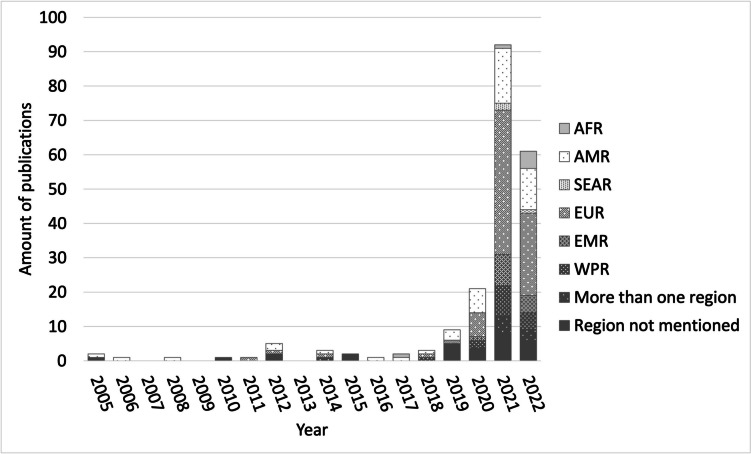
Publications per year and WHO region. Note: WHO regions are classified in African Region (AFR), Region of the Americans (AMR), South-East Asian Region (SEAR), European Region (EUR), Eastern Mediterranean Region (EMR), Western Pacific Region (WPR)

The first identified article was published in 2005 (Table S1 and Fig. 2). Research interest increased sharply in 2019 (9/205) and 2020 (21/205). The majority of articles were published in 2021 (92/205) and 2022 (61/205).

Articles were mostly focused on one specific vaccine at a time. Most of the articles investigated conspiracy narratives regarding COVID-19 vaccines (149/205). Articles about childhood vaccination were rather rare (9/205); the same held for vaccines against human papillomavirus (HPV) (13/205), the Measles, Mumps and Rubella (MMR) vaccine (5/205), or other vaccines (11/205). Additionally, we found 15/205 articles with a focus on vaccination in general instead of a specific vaccine.

We documented whether researchers used common open science practices that enable replication of findings [31, 32]. Of the 205 articles, 43 offer data sharing upon request. For 36 articles, the data is available online. For six studies, a preregistration was conducted, and additionally, five articles provide both preregistrations and open data. The remaining 125 articles do not include a statement about data availability.

### Occurrence of vaccine-related conspiracy narratives on the internet

To assess the occurrence of vaccine-related conspiracy narratives on the internet, we reviewed a total of 55 articles on these narratives. From these, 44 articles that focused on conspiracy narratives on social media outlets like Twitter; eight articles examined the occurrence of search inquiries on engines like Google; two articles investigated articles in newspapers; and one article examined the occurrence of conspiracy narratives in a movie. Across the studies different sampling methods were used (e.g., searching for vaccine-related keywords and analysing the results; analysing all comments below a specific post; analysing a set of anti-vaccination websites, etc.).

A large quantity of articles focused on conspiracy narratives about COVID-19 vaccines (25/55) [22, 23, 33–55] (Table 1). Two articles reported occurrence rates for websites or profiles propagating conspiracy narratives: 9% of the 2000 most active vaccine-related accounts on Twitter [49] and 9% of 637 analysed vaccine-related sites on several search engines and social media platforms [36]. The identified occurrence of video content with conspiracy narratives about COVID-19 vaccines showed a large range. While one article identified 2% of 118 analysed vaccine-related YouTube videos as conspiracy narratives [22], another indicated 78.9% of over 2,000 tweeted vaccine-related YouTube videos as content with conspiracy narratives [23].

**Table 1:** Overview of articles reporting prevalence of vaccine related conspiracy narratives on the internet

**COVID-19**		**General**		**Other vaccines**	
Percentage of sites/profiles sharing conspiracy narratives	Percentage of content including conspiracy narratives	Percentage of sites/profiles sharing conspiracy narratives	Percentage of content including conspiracy narratives	Percentage of sites/profiles sharing conspiracy narratives	Percentage of content including conspiracy narratives
9% [26]	2% [22]	30% [56]	2.4% [57]	2% [58]	3.8% - 14.4% [59]
9% [49]	4.9% [41]		3.5% [60]		8.7% [61]
	14.6% [45]		7% - 20% [62]		9% [63]
	15% [46] 15.1% [33]		21.3% [56]		10.5% [64]
	16.4% [44]		27.7% [65]		56.3% [66]
	16.7% [38]		41% [67]		
	19.46% - 24.68% [41]		45% [68]		
	23.5% [51]		76% [69]		
	40% [43]		100% [70]		
	78.9% [23]				

Fifteen of the 55 articles investigated the occurrence of conspiracy narratives regarding vaccination in general [56, 60, 62, 65, 67–77] (Table 1). Here, one study categorised 30% of the 204 assessed Facebook profiles expressing vaccine opposition as pages with conspiracy narratives [56]. Xu (2019) identified 2.4% of over 900 anti-vaccine articles found via Google as containing conspiracy narratives [72]. The results from Kata (2010) indicated that all of the eight analysed anti-vaccination websites presented conspiracy narratives [70].

Of the reviewed research, seven articles focused on the HPV vaccine [58, 61, 64, 66, 78–80] (Table 1). Madden et al. (2012) reported that 2% of the 89 analysed vaccine-related websites found via search engines made reference to such conspiracy narratives [58]. The occurrence of identified conspiracy narratives ranged from 8.7% (in a sample of 172 YouTube videos related to the HPV vaccine) [61] to 56.3% (in a sample of 580 posts on Instagram with related hashtags) [66].

The remaining articles referred to childhood vaccination in general [81–83], MMR [63, 84], flu [59], and tetanus [85] (Table 1). Here, every article identified conspiracy narratives as a topic in the analysed formats. For example, Nicholson and Leask (2012) identified 9% from a sample of 466 posts in online discussion forums about MMR vaccination as conspiracy narratives [63].

### Prevalence of belief in vaccine-related conspiracy narratives

Thirty-seven articles investigated the prevalence of belief in vaccine-related conspiracy narratives. Most of these studies conducted cross-sectional surveys (33/37) with samples of the general population. In sum, the 33 cross-sectional surveys had a total sample size of N = 44,630, with the smallest sample being N = 113 and the largest N = 5,822. The remaining four articles include qualitative data from a total sample of N = 305. These included data from more specific samples, e.g., parents of girls.

Overall, 29 of 33 correlative studies investigated the prevalence of belief in COVID-19 vaccine-related conspiracy narratives [24, 25, 30, 86–108] (Table S2). Among these correlative studies, the majority of authors used ad-hoc scales to measure belief in specific, COVID-19-related conspiracy narratives. Some items refer to similar narratives and thus can be compared across studies. For example, eight studies assessed the agreement with the conspiracy narrative that the pharmaceutical industry created the coronavirus to increase sales of their vaccines [86, 87, 99, 104, 105, 107]. The reported proportion of participants agreeing ranged from 5% in two studies with samples from the UK [86, Study 2 and Study 3] to 72% in a sample from Saudi Arabia [99]. Further, five studies investigated the belief in a link between the COVID-19 vaccination and infertility [30, 89, 96, 104, 105]. While 7% of a sample from Hungary agreed to this narrative [104], the agreement to this narrative was higher in a sample from Jordan (i.e., 45%) [89]. Yet, some ratings are less suitable for comparisons, as they are referring to very specific conspiracy narratives (e.g., “World superpowers use it as cover to launch a vaccination program to facilitate a global surveillance regime and establish one world order” [98]). Thus, the proportion of participants agreeing ranged from 5% (“No sense to get the vaccine, higher power manipulates health outcomes”) in a sample from Lebanon [100] to 51.7% (“The pharmaceutical industry created the COVID-19 virus “) in a sample from the US-Mexico border region [25]. In sum, the proportion of participants believing in single conspiracy narratives varied between studies. Additionally, of these 29 studies investigating the agreement with COVID-19-related conspiracy narratives, three used an adapted version of the Vaccine Conspiracy Beliefs Scale (VCBS) [92, 94, 103] developed and validated by Shapiro et al. in 2016 [109] (Table 2). A comparison of the data from the studies using the VCBS shows that they reported similar agreement ratings. For example, 14.8% of a sample from Saudi Arabia [103], 13.7% of a sample from Australia [92] and 30% of a Spanish-speaking subsample from El Salvador [94] agreed to the narrative “Vaccines are harmful and this fact is hidden”.

**Table 2:** Percentage of participants believing conspiracy narratives within articles using the Vaccine Conspiracy Beliefs Scale (VCBS)

	Fadhel (2021) [103]	Pickles et al. (2022) [92]	Caycho-Rodríguez et al. (2022) [94]
Vaccine safety data are often fabricated	29.9%	21.7%	24.7%—45.2%
Vaccines are harmful and this fact is hidden	14.5%	13.7%	4.8%—30%
Pharmaceutical companies cover up the dangers of vaccines	31%	27.9%	17.5%—36.9%
People are deceived about vaccine efficacy	26.3%	31.8%	16.5%—37.6%
Vaccine efficacy data are often fabricated	26.5%	21.2%	15%—32.3%
People are deceived about vaccine safety	25.4%	29.6%	14.6%—38.3%
The government is trying to cover up the link between vaccines and autism	*Not measured*	15%	12%—36.8%

Note: Table presents agreement to conspiracy narratives measured with the VBCS for the three studies using the scale. Reported items were adopted from the VBCS [103]. Agreement which is reported in range combined displays agreements of different subsamples

Two correlative studies provided information about the proportion of people believing at least one conspiracy narrative about the COVID-19 vaccines [97, 106]. In a Russian sample, 23% of participants supported one or more conspiracy narratives regarding the Russian COVID-19 vaccination campaign [106], and in a US sample, 33% of participants reported agreeing with one or more conspiracy narratives [97]. Additionally, two qualitative studies reported belief in conspiracy narratives as a common theme in interviews about the COVID-19 vaccines [110, 111].

The other articles (6/37) investigated the belief in conspiracy narratives focusing on other vaccines, such as against HIV and HPV (Table S2). Here, four articles examined belief in conspiracy narratives that are related to a hypothetical vaccination against HIV or a link between vaccination and HIV [112–115]. The proportion of participants believing specific conspiracy narratives ranged from 2% of participants from South Africa believing that HIV originated from vaccines [113] to 35% of participants from the USA believing that an effective HIV vaccine already exists but has been withheld from the public [114]. Additionally, one study from Texas with a sample of people with an HIV diagnosis reported that 63% of these participants believed at least one HIV-vaccine-related conspiracy narrative [115]. One qualitative study identified the belief that HIV vaccines already exist but are being suppressed as a common theme in interviews [112]. Another study investigated belief in conspiracy narratives about H1N1 among medical school students and showed that 16% believed “the whole story was a conspiracy” [116]. Furthermore, a qualitative study exploring the decline of HPV vaccination in Romania identified conspiracy narratives about HPV vaccines as a relevant topic within the interviews [117].

### Association of belief in conspiracy narratives and vaccination intention

In all, 130 articles reported 138 studies on the association between belief in conspiracy narratives and vaccination intention or vaccination uptake. We differentiate between experiments (7/138), correlative studies (122/138), and qualitative studies (9/138).

Most studies were conducted with regard to COVID-19 vaccines. Based on our search, we did not identify any experiments assessing causal relations between exposure to conspiracy narratives and COVID-19 vaccination intentions or uptake. However, 106 correlative studies investigated the association between belief in conspiracy narratives and vaccination uptake or intention, with a total sample of N = 215,646. Most sampled from the general population, with some exceptions (e.g., health care professionals [118, 119]; unvaccinated students [120]). The studies focused on different aspects of conspiracy beliefs. Most of them measured belief in COVID-19-related conspiracy narratives (70/106), followed by belief in COVID-19-vaccine-related conspiracy narratives (23/106) and a general tendency to believe in conspiracy narratives—a conspiracy mentality (21/106). Some studies included more than one kind of conspiracy belief.

Of these 106 correlative studies, 105 reported a significant negative association between belief in COVID-19-related conspiracy narratives, vaccine-related conspiracy narratives, or conspiracy mentality and vaccination intention or uptake [25, 30, 57, 86, 87, 87, 89–91, 95–98, 100, 101, 103–105, 108, 118–191] (Table 3). For example, Kaspar and Nordmeyer (2022) showed a significant negative association between conspiracy mentality and COVID-19 vaccination intention in a German sample [160], and Juanchich et al. (2021) indicated in two studies that COVID-19-related conspiracy beliefs were related to a lower vaccination intention in a sample from the UK [87]. Only seven of the 106 studies did not report significant associations between the two concepts [173, 192–197], and two showed the opposite pattern with a positive association between belief in COVID-19-related conspiracy narratives and vaccination intention [198, 199].

**Table 3:** Overview of quantity of articles reporting associations between conspiracy narratives and vaccination intention or uptake

	**COVID**	**Other vaccines**
**Experiments**		
Studies	-	7
N	-	2,438
Sample		
General public	-	7 (N = 2,438)
Results		
Effect of conspiracy narratives on Vaccination intention	-	6
No effect	-	1
**Correlative studies**		
Studies	106	13
N	215,646	140,44
Sample		
General public [studies (N)]	97 (N = 188,775)	13 (N = 14,044)
Medical staff [studies (N)]	4 (N = 14,883)	-
Non vaccinated participants [studies (N)]	5 (N = 11,988)	-
Results		
Negative associations		
Vaccination intention and belief in vaccine-related conspiracy narratives	78	7
Vaccination intention and conspiracy mentality	21	1
Vaccination uptake and belief in vaccine-related conspiracy narratives	6	2
Vaccination uptake and conspiracy mentality	-	2
*sum*	105	12
Other results		
Not associated	7	1
Positive association of vaccination intention and belief in vaccine-related conspiracy narratives	2	-
*sum*	9	1
**Qualitative studies**		
Studies	7	2
N	13,477	57
Sample		
General public	3 (N = 259)	2 (N = 57)
Medical stuff	2 (N = 1,553)	-
Non vaccinated participants	2 (N = 11,665)	-
Results		
Conspiracy narratives as identified reason for		
Vaccination refusal (uptake)	2	2
Vaccination refusal (intention)	5	-
*sum*	7	2

Additionally, seven studies used qualitative methods to investigate the relationship between belief in conspiracy narratives and vaccine intention or uptake [110, 200–205]. All studies identified belief in conspiracy narratives as a reason for vaccination refusal.

Focusing on other vaccines like childhood vaccination or vaccination in general, the reviewed literature includes seven experimental studies with a total sample size of N = 2,438, 13 correlative studies with a total sample size of N = 14,044, and two qualitative studies with a total sample size of N = 57 (Table 3).

Seven experiments provided information about the causal relationship between exposure to conspiracy narratives and vaccination intention. While Lyons et al. (2019) did not find a statistically significant effect of exposure to conspiracy narratives on vaccination intention [7], the other six experiments provided statistically significant negative effects of exposure to conspiracy narratives on vaccination intention [6, 8, 206–208]. For example, Chen et al. (2021) showed that participants exposed to vaccine-related conspiracy narratives were more hesitant towards vaccination against HPV compared to a control group [208]. Five of these seven experiments reported effect sizes. Most indicated small effect sizes, ranging from η^2^ = 0.01 [208] to η^2^ = 0.07 [206]. Only Pluviano et al. (2022) reported large effect sizes for the effect of exposure to conspiracy narratives on the intention to get a vaccination against measles (ηp^2^ = 0.674) and the Zika virus (ηp^2^ = 0.32) when comparing to a control group [207].

Of the 13 correlative studies, 12 indicated a negative association between belief in vaccine-related conspiracy narratives or conspiracy mentality and vaccination intention or uptake [6, 109, 209–217]. For example, Fonseca et al. (2019) showed a negative association between belief in vaccine-related conspiracy narratives and the intention to vaccinate their children in a sample of Portuguese parents [213], and Milošević Đorđević et al. (2021) indicated a negative relation between belief in vaccine-related conspiracy narratives and vaccination intention in Serbia [209]. One study did not find a significant association between belief in vaccine-related conspiracy narratives and vaccination intention [218].

Moreover, two studies using qualitative methods identified belief in pharmaceutical conspiracy narratives as a reason for the refusal of childhood vaccinations [219] and belief in the conspiracy narrative of polio vaccination as a Jewish plot as a reason for the refusal of the polio vaccination [220].

### Interventions to reduce the impact of vaccine-related conspiracy narratives

Finally, ten articles with 12 studies in total and a total sample of N = 18,040 evaluated interventions to reduce the impact of vaccine-related conspiracy narratives [7, 8, 182, 206, 207, 221–225] (Table 4). The tested interventions were developed either to reduce belief in conspiracy narratives (6/12) or to improve vaccine intention when vaccine-related conspiracy narratives are believed (6/12).

**Table 4:** Overview of quantity of articles tested interventions to reduce the impact of vaccine related conspiracy narratives

	**Debunking**	**Prebunking**	**Health literacy**	**Transparent communication**	**Fact checking labels**	**Social norm feedback**
Studies	5	2	1	2	1	1
N	2,218	435	1,448	13,791	206	202
**Quantity of studies that identified an effect on**						
Belief in vaccine related conspiracy narratives	1			1	1	1
Vaccination intention	3					
Vaccination intention mediated by belief in conspiracy narratives		2				
No effect	1		1	1		

Note: The table presents the quantity of articles tested interventions to reduce the impact of vaccine related conspiracy narratives and their identified effects. The tested interventions includes: Debunking (i.e., presenting counterarguments after the exposure to conspiracy narratives [223]), Prebunking (i.e., presenting counterarguments before the exposure to conspiracy narratives [206]), Health literacy (i.e., the ability to access, understand, appraise, and apply health information [221]), Transparent communication (i.e., communicating certainties and uncertainties [182], Fact checking labels (i.e., a label on social media that labels the fallacy of misinformation [225]) and Social norm feedback (i.e., information about appropriate social behaviour [222])

From six studies investigating the effect on conspiracy beliefs, four indicated a lower belief in vaccine-related conspiracy narratives within the intervention group. Here, effective interventions included social norm feedback [222], transparent communication [182], and fact-checking labels (for definitions of the interventions, see Table 4, Note) [225]. One study showed effects from a debunking text with two additional paragraphs that refer to the motives of the conspiracists and the fallacy in thinking but no effects from a more simple debunking text [223]. While the interventions reduced conspiracy beliefs, in three of the studies there was no effect on subsequent vaccination intention [222, 223, 225]. Interventions improving COVID-19 health literacy [221] or using correction messages after exposure to conspiracy narratives (i.e., debunking) [7] did not have a significant impact on belief in conspiracy narratives.

The remaining six articles focused on effects on vaccination intention [8, 182, 206, 207, 224]. Here, only Petersen et al. did not find a significant impact of transparent communication on vaccination intention [182]. Otherwise, the other interventions, namely refutational messages [8] and correction messages [207, 224] as debunking interventions and anti-conspiracy arguments prior to exposure to conspiracy narratives (i.e., prebunking; for definitions of the interventions, see Note below Table 4) [206], led to an improvement in vaccination intention compared to condition groups without an intervention. Furthermore, Jolley and Douglas (2017) found that the effect of prebunking on vaccination intention is mediated by conspiracy beliefs and the perceived danger of vaccination [206].

Four studies reported effect sizes to determine the effect of the intervention using eta squares [206, 222, 223]. All indicated small effect sizes, ranging from η^2^ = 0.03 [222] to η^2^ = 0.07 [206].

## Discussion

The present review summarises literature about i) the occurrence of vaccine-related conspiracy narratives on the internet, ii) the prevalence of belief in vaccine-related conspiracy narratives, iii) the impact of belief in conspiracy narratives on vaccination intentions and uptake, and iv) interventions to reduce the impact of conspiracy narratives.

First, the review shows that conspiracy narratives are a common type of content when searching online about (anti-)vaccination topics. Yet, we could not find a consistent pattern, as the reported percentages of sites or profiles sharing conspiracy narratives ranged from 2% [58] to over 50% [66], and the percentage of content including conspiracy narratives ranged from 2% [22] to 100% [70]. These variances might depend on the amount of analysed data, the web content and the search strategy; while some authors analysed vaccine-related content, others searched directly for anti-vaccination content. It is logical that a sample of only anti-vaccination sources more often included conspiracy narratives compared to a sample of sources that also included public health websites. Additionally, data on the prevalence of belief in conspiracy narratives provides an inconsistent picture. In particular, studies about COVID-19-related conspiracy narratives reported proportions of agreement within high ranges. This could be explained by samples from different nations and different pandemic stages where surveys were conducted. Moreover, only a minority of studies used an established scale to measure belief in conspiracy narratives, making a comparison possible at all. Thus, we focused on agreement with single conspiracy narratives instead of comparing the mean values of scales, which might reduce the variance between studies. Studies with regard to other vaccines also provided an inconsistent picture, with conspiracy narratives with a low agreement of 2% and such narratives with an agreement over 35%. In summary, there are different vaccine-related conspiracy narratives, which are believed to varying degrees. Yet, some vaccination-related conspiracy narratives are widely spread in many nations. For example, the conspiracy narratives claiming that vaccines cause autism were held as true by up to 60% of participants surveyed [25].

Further, this review provides a consistent picture of the association between conspiracy belief and vaccination intention or uptake. Across the investigated vaccines, belief in specific conspiracy narratives or a general conspiracy mentality was related to lower vaccination intention and lower vaccination uptake in nearly all the included studies. Additionally, some experiments provided information about the causality of the effect and showed that exposure to conspiracy narratives has a negative but mostly small effect on vaccination intention. However, the main part of the literature included correlative studies. Thus, statements about the causality of the effect are possible only to a limited extent. Experiments about this relationship are rare and just focus only on the effect of conspiracy beliefs on vaccination intention. Nonetheless, it can be assumed that attitudes towards vaccinations influence conspiracy beliefs, for example, when these narratives are used to justify an already-existing vaccine hesitancy.

In the review, we also collected literature about evaluated interventions that reduce the impact of conspiracy narratives in a vaccination-related context. While some interventions had an influence on conspiracy beliefs but did not influence vaccination (e.g., social norm feedback [222] and fact-checking labels [225]), there were also interventions that effectively increased vaccination intention (e.g., refutational messages [8] and correction messages [224]). These effects appear to be rather small, and therefore, it is questionable whether their extensive implementation would be worthwhile. However, the effect of exposure to conspiracy narratives on vaccination intention is also small, and thus these could indeed effectively be reduced by the investigated interventions.

Lastly, some of the reviewed articles investigated other factors that are associated with conspiracy beliefs. For example, Sallam et al. (2021) reported that higher belief in vaccine-related conspiracy narratives goes along with being female, lower education levels, and lower income [30]. Next to demographical variables, some psychosocial factors are associated with conspiracy beliefs. For example, lower levels of governmental trust [143], powerlessness [141], and higher threat perception [138] are related to conspiracy beliefs. Yet, as we did not systematically search for this research question, we decided to omit this issue.

### Limitations

While this review provides a comprehensive overview of the research about conspiracy beliefs and vaccination intention and behaviour, it has limitations. As it did not include meta-analyses, sophisticated statements about effect sizes are not possible. Moreover, we did not check for publication bias, which could lead to an overestimation of the reported effects of exposure to conspiracy narratives and of the interventions.

Additionally, we cannot guarantee that all relevant studies were included. Even if we used a rather broad search string and a selection process carried out by two raters, it can be assumed that the search strategy nevertheless missed some literature because it was not available in the used databases or in the searched languages. For example, research from the underrepresented regions could be published in other languages or in regional journals, which may not be represented in the searched databases. It is also possible that some articles investigated conspiracy narratives within a scale to measure misinformation. Since our search string only focused on conspiracy narratives only, this literature could be missing.

The review included only ten articles that evaluated interventions, as we searched for articles investigating interventions in the context of conspiracy beliefs and vaccination attitudes. Other interventions tackling misinformation to reduce vaccine hesitancy might also be important in this context [226].

The investigated literature also indicated that there is no common definition of conspiracy narratives. Some of the narratives did not fulfil all the criteria of conspiracy narratives defined by Douglas and Sutton (2023) [10]. For example, a myth claiming that a vaccine made in a specific country is safer than those made in other countries [99] was labelled as a conspiracy, but it lacks parts of the definition (e.g., malevolent intention). Since we looked at agreement ratings for each conspiracy narrative separately instead of calculating means, we decided to include such myths. Yet, it needs to be mentioned that only a few of the reported narratives in Table S2 fulfil all the criteria of a conspiracy narrative. In their recently published article, Douglas and Sutton discussed the issue of unspecific definitions of conspiracy narratives and provided a detailed definition [10].

### Directions for future studies

In sum, the review shows that at least some vaccine-related conspiracy narratives are widespread and that belief in conspiracy narratives is associated with vaccine hesitancy. Moreover, the review also points out considerable gaps in the current state of research.

First, it was not possible to estimate the prevalence of vaccine-related conspiracy narratives on the internet as the samples and methods used in the reviewed studies differed too much. Thus, future research in this field should focus on more consistent and generalizable ways of quantifying the prevalence of (vaccine-related) conspiracy theories to make a comparison possible.

Future studies measuring belief in conspiracy narratives should use validated scales like the VCBS [109] to enable a comparison across studies. This would also enable a comparison of scale values instead of comparing single conspiracy narratives.

Additionally, a future systematic review could also extract demographic variables and psycho-social factors associated with believing vaccination-related conspiracy narratives for better understanding of those believing conspiracy narratives.

Numerous studies have used correlative data to investigate the relationship between conspiracy beliefs and vaccination intention within the general population. More research on specific samples could expand the current state of research. For example, as ostracism leads to higher conspiracy beliefs [227], research on social outgroups that are particularly affected by a disease or a pandemic could be interesting. Focusing on vaccination providers like physicians could also lead to new findings. In these cases, belief in conspiracy narratives could also be associated with fewer vaccination recommendations, which could influences the vaccination intention of their patients [228]. Moreover, research in non-WEIRD countries appears to be still patchy.

Additionally, to gain better knowledge about the direction of the relationship between conspiracy beliefs and vaccine hesitancy, more experiments are needed. It could be possible that vaccine hesitant individuals are more susceptible to conspiracy narratives as they justify their hesitancy. Thus, it could be possible that the concepts influence each other.

Considering the small effects of the evaluated interventions, it might be worth the effort to develop and test interventions that have greater impacts. For example, with regard to vaccination as an important health-related topic, it might be beneficial to use the high trustworthiness of health care professionals [229] and provide them with techniques for rebutting fake news and conspiracy narratives [230].

Finally, we recommend that other researchers use open science practices to improve the quality and reproducibility of behavioural science [31, 32]. Open data makes research transparent and enables other researchers to replicate the findings, and preregistration enhances the validity of results by ensuring that any subsequent adjustments made are traceable to the original data.

## Conclusion

To conclude, the results showed that many vaccine-related conspiracy narratives have spread and are believed to varying degrees. This was especially the case during the COVID-19 pandemic. Further, exposure to these conspiracy narratives can decrease vaccination intention. There are already some promising interventions that could reduce the effect of vaccine-related conspiracy narratives. However, the current state of research is largely based on correlative studies and could be strengthened with more research enabling causal interpretations.

## Supplementary Information

Supplementary Material 1.

## Data Availability

The datasets supporting the conclusions of this article are available in the Open Science Framework (OSF), https://osf.io/2z4et/.
