# Supplementary material for: Conspiracy narratives and vaccine hesitancy: a scoping review of prevalence, impact, and interventions
Source: BMC Public Health. 2024 Nov 29;24:3325. doi: 10.1186/s12889-024-20797-y (PMC11606073; doi:10.1186/s12889-024-20797-y)
Supplement: Supplementary file 1 — Supplementary Material 1. [file 12889_2024_20797_MOESM1_ESM.docx]

**Conspiracy Narratives and Vaccine Hesitancy: A Scoping Review of Prevalence, Impact, and Interventions**

**Supplement**

[Table S1: Publications per year and region 1](#_Toc161647291)

[Figure S1: Publications per year and region 2](#_Toc161647292)

[Table S2: Overview of articles reporting prevalence of vaccine-related conspiracy narratives 3](#_Toc161647293)

# Table S1: Publications per year and region

|  | EUR | AMR | WPR | EMR | AFR | SEAR | More than one region | Region not mentioned | Sum |
| --- | --- | --- | --- | --- | --- | --- | --- | --- | --- |
| 2022 | 24 | 12 | 5 | 5 | 5 | 1 | 3 | 6 | 61 |
| 2021 | 42 | 16 | 9 | 9 | 1 | 2 | 5 | 8 | 92 |
| 2020 | 7 | 7 | 2 | 1 | 0 | 0 | 1 | 3 | 21 |
| 2019 | 1 | 3 | 0 | 0 | 0 | 0 | 0 | 5 | 9 |
| 2018 | 1 | 1 | 1 | 0 | 0 | 0 | 0 | 0 | 3 |
| 2017 | 0 | 1 | 0 | 0 | 1 | 0 | 0 | 0 | 2 |
| 2016 | 0 | 1 | 0 | 0 | 0 | 0 | 0 | 0 | 1 |
| 2015 | 0 | 0 | 0 | 0 | 0 | 0 | 0 | 2 | 2 |
| 2014 | 1 | 1 | 0 | 0 | 0 | 0 | 1 | 0 | 3 |
| 2013 | 0 | 0 | 0 | 0 | 0 | 0 | 0 | 0 | 0 |
| 2012 | 1 | 2 | 0 | 0 | 0 | 0 | 0 | 2 | 5 |
| 2011 | 1 | 0 | 0 | 0 | 0 | 0 | 0 | 0 | 1 |
| 2010 | 0 | 0 | 0 | 0 | 0 | 0 | 0 | 1 | 1 |
| 2009 | 0 | 0 | 0 | 0 | 0 | 0 | 0 | 0 | 0 |
| 2008 | 0 | 1 | 0 | 0 | 0 | 0 | 0 | 0 | 1 |
| 2007 | 0 | 0 | 0 | 0 | 0 | 0 | 0 | 0 | 0 |
| 2006 | 0 | 1 | 0 | 0 | 0 | 0 | 0 | 0 | 1 |
| 2005 | 0 | 1 | 0 | 0 | 0 | 0 | 0 | 1 | 2 |
| Sum | 78 | 47 | 17 | 15 | 7 | 3 | 10 | 28 | 205 |

Note: Table presents the number of identified publications grouped by WHO region and year of publication. Publications with data conducted in more than one WHO region are labelled as “More than one region”. Publications without any information about the region are labelled as “Region not mentioned”.

# Figure S1: Publications per year and region


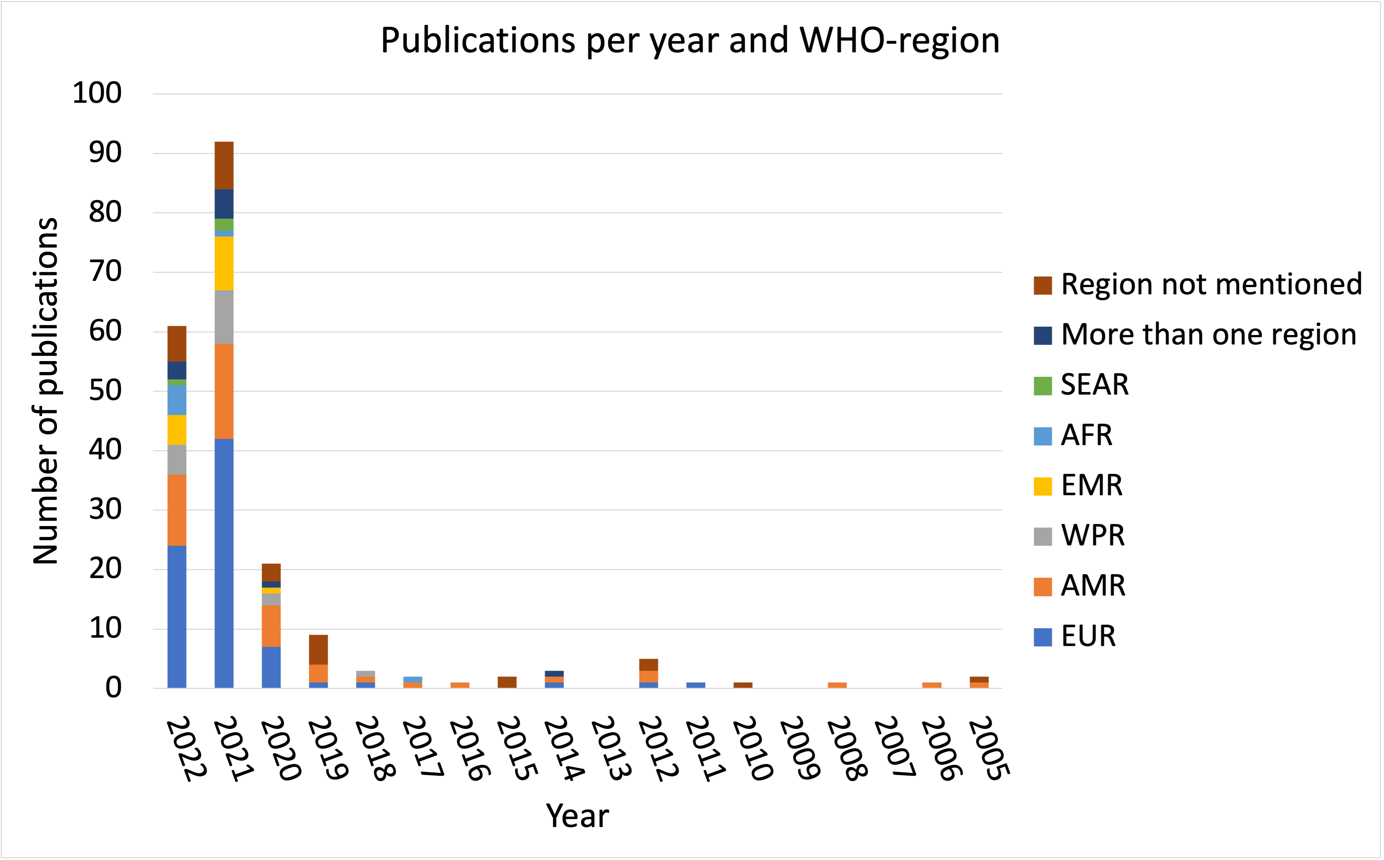


Note: Figure presents the number of identified publications grouped by WHO region and year of publication. Publications with data conducted in more than one WHO region are labelled as “More than one region”. Publications without any information about the region are labelled as “Region not mentioned”.

# Table S2: Overview of articles reporting prevalence of vaccine-related conspiracy narratives

| Author | Year | Nation | Topic | Items | Agreement |
| --- | --- | --- | --- | --- | --- |
| Priddy et al. | 2006 | USA | HIV | *Self-developed scale:*  An effective HIV vaccine already exists but has been withheld from the public. | 35% |
| Mavros et al. | 2011 | Greek | H1N1 | *Self-developed scale:*  I believe the whole story is a conspiracy. | 16% |
| Hogg et al. | 2017 | South Africa | HIV | *Self-developed scale:*  HIV is originated from vaccines. | 2% |
| Romer & Jamieson | 2020 | US | Covid-19 | *Self-developed scale:*  The pharmaceutical industry created the coronavirus to increase sales of its drugs and vaccines. | 14.8% |
| Freeman et al. | 2020 | UK | Covid-19 | *OCEANS coronavirus conspiracy scale:* Big Pharma created COVID-19 to profit from the vaccines. COVID-19 was created to force everyone to get vaccinated. The vaccine will be used to carry out mass sterilisation. WHO already has a vaccine and are withholding it. | 10.6%  11.7%  11.4%  10.5% |
| Altenbuchner et al. | 2021 | Germany | Vaccination in general | *Self-developed scale:*  Pharmaceutical companies play down the dangers of vaccines.  Side effects of vaccinations are often concealed.  The effectiveness of vaccines is often exaggerated.  Attempts are made to cover up the link between vaccines and autism. | 56.9%  51.5%  34.5  19.5% |
| Juanchich et al.  Study 1 | 2021 | UK | Covid-19 | *Self-developed scale:*  The new coronavirus was part of an effort by pharmaceutical companies to profit by selling vaccines for it. | 7% |
| Juanchich et al.  Study 2 | 2021 | UK | Covid-19 | *Self-developed scale:*  The new coronavirus was part of an effort by pharmaceutical companies to profit by selling vaccines for it.  There is a vaccine or cure for the coronavirus that the government won't release/authorise. | 5%  9% |
| Juanchich et al.  Study 3 | 2021 | UK | Covid-19 | *Self-developed scale:*  The new coronavirus was part of an effort by pharmaceutical companies to profit by selling vaccines for it.  There is a vaccine or cure for the coronavirus that the government won't release/authorise. | 5 – 7%  8% |
| Nowak et al. | 2021 | Poland | Covid-19 | *Self-developed scale:*  COVID-19 pandemic as an action of pharmaceutical industry to sell vaccines. | 27.2% |
| Tsantopoulos et al. | 2021 | Greece | Covid-19 | *Self-developed scale:*  COVID-19 is a manufactured virus for which a vaccine had been developed before it emerged. | 38.2 – 48.1% |
| Jensen et al. | 2021 | Germany | Covid-19 | *Self-developed scale:*  The coronavirus (COVID-19) is part of a global effort to enforce mandatory vaccination. | 15% |
| Sallam et al. | 2021 | Jordan | Covid-19 | *Self-developed scale:*  COVID-19 was man-made to enforce vaccination. COVID-19 vaccination will be used to implant microchips into humans to control them. COVID-19 vaccination can lead to infertility. | 11%  26.4%  45.4% |
| Yang et al. | 2021 | China | Covid-19 | *Self-developed scale:*  COVID-19 vaccine effectiveness data are often fabricated by pharmaceutical companies. The government often conceals the safety deficiencies of vaccines. Pharmaceutical companies cover up the danger of vaccines.  People are often deceived about the effectiveness of the vaccines. The fact that vaccines are harmful for children is deliberately obscured. | 19.5%  16.7%  17.4%  18.9%  14.2% |
| Sallam et al. | 2021 | Jordan, Kuwait and other Arab countries | Covid-19 | *Self-developed scale:*  The current coronavirus was man-made to force everyone to get vaccinated. Coronavirus vaccine will be a way of implanting people with microchips to control humans.  COVID-19 vaccines will lead to infertility | 40%  27.2%  23.4% |
| Szmyd et al. | 2021 | Poland | Covid-19 | *Self-developed scale:*  Mircochip injection  Limitation of civil rights  Control of births  Autism | 1.8 – 5.2%  2.5 – 11.7%  0.7 – 4%  4 – 5.5% |
| Strathdee et al. | 2021 | US-Mexico border region | Covid-19 | *Self-developed scale:*  Thinks that the pharmaceutical industry created the COVID-19 virus. Thinks that vaccines given to children for diseases like measles and mumps cause autism. Thinks that COVID vaccines being offered to “people like me” are not as safe as other COVID vaccines. Thinks that COVID vaccines include a tracking device. Thinks that some COVID vaccines could change their DNA. | 51.7%  63.6%  40.7%  34.4%  30.8% |
| Arshad et al. | 2021 | Pakistan | Covid-19 | *Self-developed scale:*  The COVID-19 vaccine has safety issues, which can kill people. The COVID-19 vaccine contains any 5G Nano-chips to control people. The COVID-19 vaccine could take away reproducibility (or cause infertility). COVID-19 and its vaccine are created to control the world population. COVID-19 and its vaccine is designed to harm the Muslim nations. The COVID-19 vaccine can harm people’s health as this has been developed in a very short period?  COVID-19 and its vaccine are non-Muslims’ propaganda to rule the world? | 19%  9.3%  12%  18.4%  13.1%  8.4%  16.4% |
| Alrajeh et al. | 2021 | Saudi Arabia | Covid-19 | *Self-developed scale:*  Pharmaceutical companies are encouraging the spread of coronavirus to make a profit through selling vaccine. The coronavirus is a myth to force vaccinations on people. Drug companies cover up the side effects of vaccines. People are deceived about the effectiveness of vaccines. COVID-19 vaccine can result in autism. A coronavirus vaccination could give one coronavirus. COVID-19 vaccines made in America and Europe are safer than those made in other countries. COVID-19 vaccines made in China and Russia are safer than those made in other countries.  COVID-19 vaccines made in India are safer than those made in other countries. | 72.3%  55.5%  77.5%  72%  29.5%  49%  12%  6.5%  2% |
| Bou Hamdan et al. | 2021 | Lebanon | Covid-19 | *Self-developed scale:*  Pharmaceutical companies have hidden information about the vaccine’s bad health outcomes. No sense to get the vaccine, higher power manipulates health outcomes. COVID-19 vaccine is attempt to take away personal freedom. Government is using the vaccine to control population. | 10%  5%  6%  8% |
| Fadhel | 2021 | Saudi Arabia | Covid-19 | *Vaccine Conspiracy Beliefs Scale (VCBS):* Vaccine safety data are often fabricated. Vaccines are harmful and this fact is hidden. Pharmaceutical companies cover up the dangers of vaccines. People are deceived about vaccine efficacy. Vaccine efficacy data are often fabricated. People are deceived about vaccine safety. | 29.9%  14.5%  31%  26.3%  26.5%  25.4% |
| Altenbuchner et al. | 2021 | Germany | Covid-19 | *Self-developed scale:*  Pharmaceutical companies play down the dangers of vaccines. Side effects of vaccines are often concealed. The effectiveness of vaccines is often exaggerated. Attempts are made to cover up a link between vaccines and autism. | 56.9%  51.5%  34.5%  19.5% |
| McCarthy et al. | 2022 | Australia | Covid-19 | *Self-developed scale:*  COVID-19 vaccines will be used to harm or control society. | 34.9% |
| Pickles et al. | 2022 | Australia | Covid-19 | *Vaccine Conspiracy Beliefs Scale (VCBS):*  Data about vaccine safety is often fabricated (made up). People are deceived about the effectiveness of vaccines. Immunising is harmful, and this fact is covered up. Drug companies cover up the dangers of vaccines. Data about vaccine effectiveness is often fabricated (made up). People are deceived about vaccine safety. The government is trying to cover up the link between vaccines and autism. | 21.7%  31.8%  13.7%  27.9%  21.2%  29.6%  15% |
| Zaidi et al. | 2022 | Pakistan | Covid-19 | *Self-developed scale:*  COVID-19 vaccine was a conspiracy of the West. | 17% |
| Caycho-Rodríguez et al. | 2022 | 13 Spanish-speaking countries | Covid-19 | *Vaccine Conspiracy Beliefs Scale (VCBS):*  COVID-19 Vaccine safety data is often fabricated. Vaccinating children against COVID-19 is harmful and this fact is covered up. Pharmaceutical companies cover up the dangers of COVID-19 vaccines. People are deceived about COVID-19 vaccine efficacy. COVID-19 Vaccine efficacy data is often fabricated. People are deceived about COVID-19 vaccine safety. The government is trying to hide the link between COVID-19 vaccines and the appearance of other diseases. | 24.7 – 45.2%  4.8 – 30%  17.5 – 36.9%  16.5 – 37.6%  15% - 32.3%  14.6 – 38.3%  12 – 36.8% |
| Ghaddar et al. | 2022 | Lebanon | Covid-19 | *Self-developed scale:*  COVID-19 is business tool to sell vaccines and medicaments. | 33% |
| Jamil et al. | 2022 | Pakistan | Covid-19 | *Self-developed scale:*  World superpowers use it as cover to launch a vaccination program to facilitate a global surveillance regime and establish one world order. | 38.1% |
| Ahiakpa et al. | 2022 | Africa | Covid-19 | *Self-developed scale:*  The COVID-19 vaccine will alter your DNA. The COVID-19 vaccine contains a tracking device. The COVID-19 vaccine for Africa is different from that in other continents. | 14.8%  11%  32.6% |
| Hammad et al. | 2022 | Jordanian | Covid-19 | *Self-developed scale:*  The COVID-19 vaccine includes a tracking device. The COVID-19 vaccine includes a microchip to control us. The vaccine was accepted after the manufacturing companies coerced the authorities. The vaccine is designed to decrease number of human populations. | 12.7%  12.5%  27%  23.5% |
| Biro-Nagy and Szaszi | 2022 | Hungary | Covid-19 | *Self-developed scale:*  With the COVID-19 vaccination, a microchip may be built in the body secretly. COVID-19 vaccination may cause infertility; the secret goal of the vaccination is population control. Coronavirus was developed by pharmaceutical companies in order to help them sell their drugs and vaccines more easily. | 5%  6%  14% |

Note: Table presents measured agreement to different conspiracy narratives. Reported items were adopted from the studies. Agreement which is reported in range combined displays agreements of different subsamples.
